# Obituary: Tim Meese

**DOI:** 10.1177/03010066261470669

**Published:** 2026-08-07

**Authors:** Nick Scott-Samuel, Derek Arnold, Mark Georgeson, Ute Leonards, George Lovell, Andrew Schofield

**Affiliations:** 1 University of Bristol, Bristol, UK; 2 University of Queensland, Brisbane, Australia; 3 1722 Aston University, Birmingham, UK; 4 Abertay University, Dundee, UK


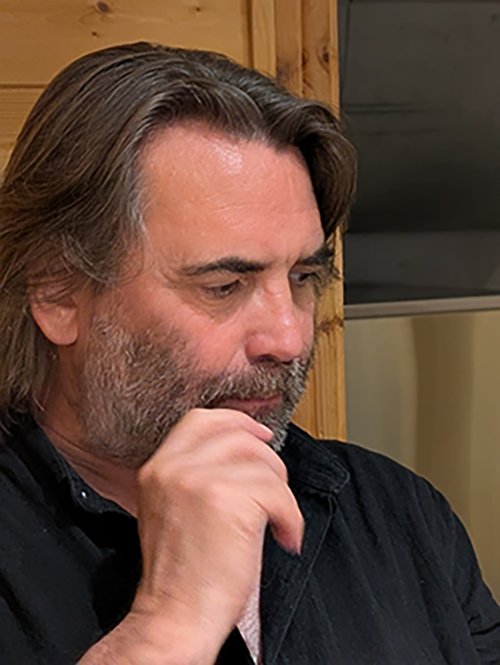
Tim Meese, who died of a heart attack at the age of 61 on 13 May 2026, has left the building prematurely. This is very much out of character for someone who was generally amongst the last people standing, whether at a conference or a party. It also jars with Tim's larger-than-life presence, and it is hard for us – his colleagues and friends – to contemplate how much less fun and intellectually stimulating future meetings will be without him.

Tim worked briefly for British Telecom before studying Computer Science and Psychology at Newcastle University, graduating with first-class honours in 1989. He received his PhD from the University of Bristol, supervised by Mark Georgeson, before moving to the University of Birmingham for a postdoc with Mike Harris. In 1996, he became a Lecturer in the Department of Vision Sciences at Aston University, where he remained for the rest of his career, progressing to Senior Lecturer in 2003, Reader in 2008 and Professor in 2011. He received his 30 years’ service award at a dinner shortly before his untimely death.

Initially an enthusiast for plaid stimuli (e.g., Meese & Georgeson, 1996), he was sidetracked by the merits of different staircase procedures during his PhD; this work expanded his thesis to two volumes and produced a methodological paper (Meese, 1995) alongside his empirical work on edges. That two-volume thesis was a terrifying sight for subsequent postgraduates, sitting menacingly on a shelf in the Georgeson lab. Tim went on to investigate various aspects of optic flow (e.g., Meese & Harris, 1997), and then around 2003 turned his attention to the mechanisms of spatial vision with two eyes. This period produced a string of highly original, seminal papers on binocular vision (e.g., Baker et al., 2007; Baker et al., 2012; Georgeson et al., 2016; Meese et al., 2006) and on summation of signal contrast across visual space (e.g., Baker & Meese, 2011; Meese & Summers, 2009; Meese & Baker, 2011) in both normal vision and amblyopia (e.g., Baker et al., 2008). These and other papers link experiments with modelling to strengthen our understanding of fundamental processes in early vision.

But Tim was also diverted at various times by higher level things such as second-order vision (Summers et al., 2015), illusions (Scott-Samuel et al., 2018), the tilt-shift effect (Meese et al., 2023), dazzle camouflage (Lovell et al., 2024), expertise in Ordnance Survey map-makers (Skog et al., 2024), how to mark an undergraduate essay (Meese & Hosie, 2026), and philosophy of perception (Meese, in press). At the time of his death, Tim was investigating the role of binocular disparity and pictorial cues to stereopsis on size perception using Aston's Virtual Reality CAVE.

Working on research projects with Tim was always a great experience. It would be no surprise to hear that he’d been digging in an archive somewhere, for example, visiting the National Archives at Kew Gardens to see if he could find a letter from Graham Kerr to Winston Churchill. His interest in obscure materials extended to his hobbies too, such as his description of Tourret's *Petroleum Rail Tank Wagons of Britain* as ‘a fine volume’. Never one to speak without thinking, he’d often fall disconcertingly silent only to come back, maybe days later, with some insightful comment. Whatever the project, he inevitably improved the design and rigour of the experiments proposed, and he was always an active writing partner. He strongly believed that it was the job of the author to make papers accessible to the reader and would spend ages deliberating over word choice and phrasing. His publication record is all the more remarkable given the care he put into every paper.

Tim applied the same rigour, thought and care to his many citizenship roles at Aston. He was director of the Aston Laboratory for Immersive Virtual Environments, a facility that he built from scratch with a mix of internal, external and charity funding. He designed Aston's immersive Virtual Reality CAVE to have both active and passive stereo projection to reduce EEG artefacts and spent many months getting that system to work as well as it could. He took on vital but ‘unseen’ administration roles such as PGR tutor for the College of Health and Life Sciences where he supported countless PhD students through difficult times, often working behind the scenes and in confidence to repair relationships.

Outside Aston, Tim devoted much time over several decades supporting the AVA (Applied Vision Association) in organising its Easter scientific meetings at different locations around the UK. Each Christmas, though, Tim arranged the Xmas AVA meetings at Aston, where the post-meeting party became legendary – until banned by those in authority.

At conferences, Tim seemed omnipresent, scrupulously attending the talk and poster sessions, yet also finding time to socialise into the small hours. For many of us, he was the first person to look for on arrival at a meeting: a quick greeting, and then on to the serious business of finding the best restaurants and bars to frequent over the next several days.

Tim cut a distinctive figure, with a quite particular dress sense: a fondness for waistcoats and linen shirts, and a more-or-less absolute refusal to wear shorts, no matter how hot the weather. His wildly waving arms were a useful cue to his friends in crowded conditions. He appreciated good food and drink, and was always up for experiencing a novel dish or new flavour. At the Asia Pacific Conference on Vision meeting (Taiwan, 2017), Tim was the only one brave enough to allow snakes to wrap around his head and shoulders, before tasting snake soup.

As a house guest, he almost always managed to leave something behind (usually a pair of glasses). Conversation with Tim never flagged and often meandered in quite unexpected directions. He had strong (and generally well informed) views on most things and displayed a touching astonishment that anyone could possibly have different opinions from him. He could talk passionately about all manner of topics, seamlessly switching from cricket to American football, punk to Eurovision, psychophysics to trains. His dancing was a thing to behold. With furrowed brow, deep in concentration, he took the same approach to it as he did to everything: systematic, idiosyncratic and enthusiastic. Tim will be sorely missed; the world is dimmer without him.

## Further Memories of Tim can be Found Here:

https://app.givetastic.com/c/messages-to-remember-professor-tim-meese
